# Dietary Salt Reduction and Cardiovascular Disease Rates in India: A Mathematical Model

**DOI:** 10.1371/journal.pone.0044037

**Published:** 2012-09-06

**Authors:** Sanjay Basu, David Stuckler, Sukumar Vellakkal, Shah Ebrahim

**Affiliations:** 1 Department of Medicine, Stanford Prevention Research Center, Stanford University, Stanford, California, United States of America; 2 Department of Public Health and Policy, London School of Hygiene and Tropical Medicine, London, United Kingdom; 3 Department of Sociology, Cambridge University, Cambridge, United Kingdom; 4 South Asia Network for Chronic Disease, Public Health Foundation of India, New Delhi, India; 5 Department of Non-Communicable Disease Epidemiology, London School of Hygiene and Tropical Medicine, London, United Kingdom; University of Queensland, Australia

## Abstract

**Background:**

Reducing salt intake has been proposed to prevent cardiovascular disease in India. We sought to determine whether salt reductions would be beneficial or feasible, given the worry that unrealistically large reductions would be required, worsening iodine deficiency and benefiting only urban subpopulations.

**Methods and Results:**

Future myocardial infarctions (MI) and strokes in India were predicted with a Markov model simulating men and women aged 40 to 69 in both urban and rural locations, incorporating the risk reduction from lower salt intake. If salt intake does not change, we expect ∼8.3 million MIs (95% CI: 6.9–9.6 million), 830,000 strokes (690,000–960,000) and 2.0 million associated deaths (1.5–2.4 million) per year among Indian adults aged 40 to 69 over the next three decades. Reducing intake by 3 g/day over 30 years (−0.1 g/year, 25% reduction) would reduce annual MIs by 350,000 (a 4.6% reduction; 95% CI: 320,000–380,000), strokes by 48,000 (−6.5%; 13,000–83,000) and deaths by 81,000 (−4.9%; 59,000–100,000) among this group. The largest decline in MIs would be among younger urban men, but the greatest number of averted strokes would be among rural men, and nearly one-third of averted strokes and one-fifth of averted MIs would be among rural women. Only under a highly pessimistic scenario would iodine deficiency increase (by <0.0001%, ∼1600 persons), since inadequate iodized salt access—not low intake of iodized salt—is the major cause of deficiency and would be unaffected by dietary salt reduction.

**Conclusions:**

Modest reductions in salt intake could substantially reduce cardiovascular disease throughout India.

## Introduction

Ischaemic heart disease (IHD) and stroke are leading causes of death in India, and hypertension is thought to be their main risk factor [1]. The World Health Organization (WHO) estimates that nearly half of these deaths occur among adults aged 30 to 69 years old [2]. The prevalence of hypertension has risen exponentially in India over the past three decades, from less than 5% of adults to over 25% of urban and 10% of rural adults today [3].

High salt intake is associated with increased risk of myocardial infarction (MI) and stroke [4], principally because salt intake significantly affects blood pressure [5], [6]. Although the WHO recommends that adults consume less than 5 grams (g) of salt per day in order to reduce hypertension-associated cardiovascular and cerebrovascular disease, Indian adults consume between 8.5 g and 15 g per day on average [7], [8] (as compared to an average 8.9 g per day among Americans [9]).

Several Western countries including the UK and Finland have implemented regulations on salt content in food, experiencing associated declines in per capita salt intake and heart attacks, strokes and deaths [10], [11], [12]. Whether and how to reduce India's high level of dietary salt intake is currently being debated [13]. Several commentators have suggested that the reductions in salt intake necessary for lowering cardiovascular mortality in developing countries like India are unrealistically large, that benefits would accrue only among urban populations exposed to Western diets, and that the rural poor would experience higher disease rates related to iodine deficiency because of inadequate intake of iodized salt [14], [15], [16], [17].

To explore these assertions, we constructed a mathematical model of MI and stroke in India.

## Methods

We constructed a Markov (state-transition) model of MI and stroke among India's population following conventional standards for decision-analytic modeling [18]. The detailed model is specified in Text S1 to permit full replication of our results; here, we outline the general structure, critical assumptions and validation of the model.

### Model structure

The model simulates Indian adults organized into twelve cohorts, defined by gender, age (grouped into 40–49, 50–59, and 60–69 year old cohorts) and location (urban versus rural) from 1998 to 2050. The age groups were limited to 40 through 69 years old because consistent prospective data from multiple Indian studies were available from which to estimate MI and stroke risk among Indian populations in these age groups [19], [20]. We used an “open cohort” approach, meaning that aging was explicitly simulated between the age groups; new entries into the 40–49 year old age class were incorporated into every time step of the simulation, based on Indian census projections (Figure S1) [21]. Urban-rural migration was not specifically modeled with a separate variable, but inferred from the Census estimates.

**Figure 1:**
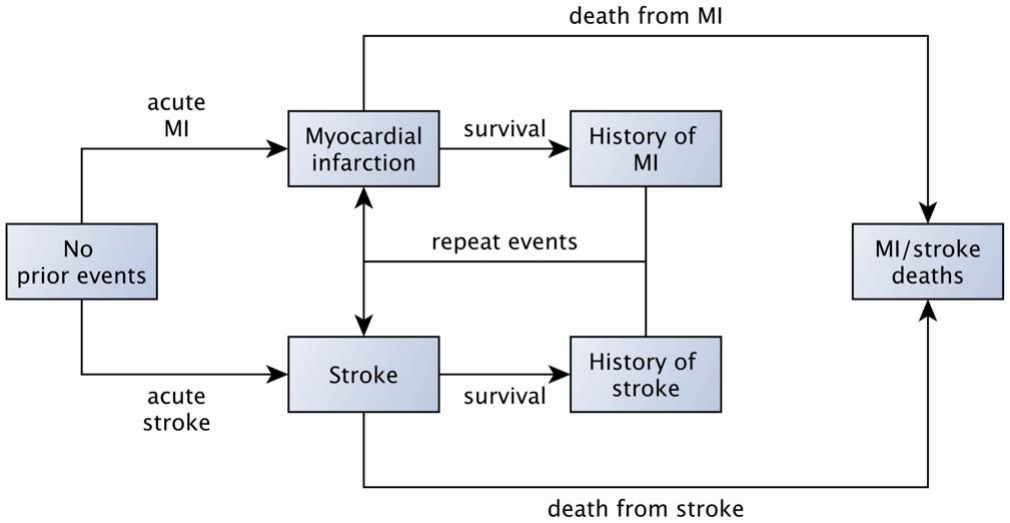
Model diagram. Health states are further divided into age-, gender- and location-specific (urban and rural) submodels. Deaths from non-cardiovascular events are calculated from each compartment of the model at each time point in the simulation (not drawn). The transition probabilities between health states in the model are detailed in Tables S1, S2 and S3. Dietary salt reduction in the model lowers the risk of incident and recurrent myocardial infarction and stroke events. MI: myocardial infarction.

In each monthly time step of the model, an individual can be in one of seven possible states, as depicted in Figure 1: a state with no history of IHD or stroke; a state of having had an acute MI during that month; a state of having had an acute stroke during that month; a state of having survived a MI; a state of having survived a stroke; a state of having died from either an MI or a stroke; and a state of having died from a non-MI/non-stroke event.

### Data sources for transition probabilities

As detailed below, the impact of reduced salt on reduced blood pressure, and reduced blood pressure on the relative risk of MI and stroke, were taken from international sources; the baseline risk of MI and stroke as well as estimates of blood pressure in each cohort were obtained from India-specific sources (see Table 1 for parameters, data and sources). Monthly risks of MI and stroke among each cohort were taken from a WHO analysis of estimates among Indian adults, organized by age, gender and urban versus rural location (Table S1) [22]. We incorporated the time-trend in these risks (Table S2), to account for how rates of MI and stroke are changing among the population.

**Table 1:** Model parameters and data sources.

*Parameter*	*Source*
Population size among cohorts - *Figure S1*	Indian Census [21]
Risk of MI or stroke (incident and recurrent) among cohorts - *Tables S1, S2*	World Health Organization [22]
Fatality rate from MI and stroke among cohorts - *Table S3*	World Health Organization [22]
Non-MI/non-stroke mortality rates among cohorts - *Tables S4, S5*	World Health Organization [24]
Hypertension prevalence among cohorts - *Table S6*	World Health Organization [43]
Iodine levels in salt among provinces – *Figure S3*	Indian National Family Health Survey [51]
Systolic blood pressure reduction for each gram salt reduction – *Text S1*	Meta-analysis [27]
Relative risk reduction in MI/stroke from each mmHg reduction in systolic blood pressure – *Text S1*	Meta-analysis [19] and mathematical model [26]

The risk of recurrent MI/stroke and death from acute MI/stroke among each cohort was also adopted from the WHO analysis (Tables S1, S2 and S3) [22]. The risk of death from non-MI/non-stroke causes was incorporated in the model using a standard competing risks algorithm [23], which applies cause-specific mortality rates (Table S4) and time-trends in non-MI/non-stroke deaths (Table S5) from the WHO to each cohort in the model [24]. To ensure face validity of the model results, we compared the model's disease-specific mortality estimates against independent estimates of mortality that had not been used as input data sources (Figure S2).

### Salt reduction simulations and underlying assumptions

To simulate the impact of salt reduction, we performed a two-stage calculation: first, given a particular hypothetical level of reduction in salt intake, we estimated how much blood pressure would be reduced (assuming a continuous linear relationship between salt reduction and blood pressure reduction, as in prior models, [25], [26]); then we estimated how much the relative risk of incident MI and stroke would be reduced by this reduction in blood pressure. We used the most conservative multi-country meta-analysis data available to estimate the reduction in systolic blood pressure from salt reduction [27]; this corresponds to a reduction of 3.6 mmHg in systolic pressure (95% CI: 2.8 to 4.4) among hypertensive persons and 1.8 mmHg (95%CI: 1.0 to 2.6) among normotensive persons from a 3 g/day decline in dietary salt intake. Blood pressure among each Indian cohort was taken from the WHO SAGE study [28], a nationally-representative household survey in which blood pressure was established by examination, using the average of three readings (Table S6). The Smith-Spangler equations were then used to estimate the age-specific relative risk reduction for incident MI and stroke from each millimeter mercury reduction in systolic pressure [26]; these equations (see Text S1) provide estimates of relative risk from a multi-national meta-analysis [19]. Based on a prior review [29], we simulated that stroke risk reduction would be achieved gradually at a linear rate over three years, while for myocardial infarctions, two-thirds of the benefit would occur at a linear rate over the first three years and the rest would occur at a linear rate over the subsequent seven years.

We considered a reduction in per capita salt intake of 3 g/person/day achieved over 30 years (at a linear rate of −0.1 g/year from the year 2013 through 2043). As described in Box S1, we additionally ran the model under varying assumptions and analyzed the risk of iodine deficiency associated with dietary salt reduction. We conducted further multivariate uncertainty analyses to produce confidence intervals around our model's results via 10,000 simulations in each scenario (Box S1), and varied the salt reduction from 1 g to 4 g (Text S1).

## Results

Without changes to current levels of dietary salt intake, our model projects that Indians in the 40 to 69 year old age group will experience an annual rate of approximately 8.3 million new and recurrent myocardial infarctions (95% CI: 6.9–9.6 million), 830,000 new and recurrent strokes (95% CI: 690,000–960,000) and 2.0 million deaths (95%CI: 1.5–2.4 million) from either cause on average during each of the next 30 years.

A population-wide reduction in dietary salt intake of 3 g/day achieved over 30 years (at a linear rate of 0.1 g/year, the rate achieved in Finland) is projected to reduce the rate of MI by about 14.6 per 10,000 (from 196.9 to 182.3 per 10,000), stroke by 2.1 per 10,000 (from 19.4 to 17.3 per 10,000), and deaths from either cause by 3.4 per 10,000 (from 47.3 to 43.9 per 10,000; Table 2). Given the projected population size of India, this amounts to preventing an annual average of approximately 350,000 MIs (95% CI: 320,000–380,000), 48,000 strokes (95% CI: 13,000–83,000), and 81,000 deaths (95% CI: 59,000–100,000). A lesser reduction of 1 g/day of dietary salt intake achieved over 30 years is projected to avert approximately 120,000 MIs (95% CI: 110,000–130,000), 16,000 strokes (95% CI: 4,500–28,000) and 27,000 deaths (95% CI: 20,000–35,000) annually, while a greater reduction to 4 g/day achieved over the same 30 year duration is expected to prevent about 460,000 MIs (95% CI: 430,000–500,000), 64,000 strokes (95% CI: 17,000–110,000) and 110,000 deaths (95% CI: 78,000–140,000) annually.

**Table 2:** Sensitivity analyses.

Outcome	Urban men	Urban women	Rural men	Rural women	Overall population
Reduction in annual rate per 10,000 persons (95% CI) *(% change in rate from 2013 to 2043)*					
Main simulation					
Incident MI	35.4+/−5.8 *(8.0%)*	13.9+/−2.3 *(7.5%)*	5.0+/−0.8 *(7.4%)*	8.4+/−1.4 *(7.6%)*	14.7+/−2.4 *(7.6%)*
Incident Stroke	2.0+/−0.3 *(11.2%)*	2.0+/−0.3 *(10.8%)*	2.1+/−0.4 *(10.2%)*	2.2+/−0.4 *(10.7%)*	2.1+/−0.4 *(10.7%)*
Deaths from either cause	8.5+/−1.9 *(8.1%)*	1.9+/−0.4 *(8.0%)*	2.3+/−0.5 *(7.5%)*	1.5+/−0.3 *(8.1%)*	3.4+/−0.8 *(7.9%)*
Lower risk reduction with blood-pressure lowering*					
Incident MI	29.6+/−7.8 *(6.7%)*	11.9+/−3.0 *(6.4%)*	4.4+/−1.1 *(6.5%)*	7.2+/−1.8 *(6.6%)*	12.4+/−3.2 *(6.6%)*
Incident Stroke	1.7+/−0.5 *(9.3%)*	1.8+/−0.5 *(9.3%)*	1.8+/0.5 *(9.0%)*	1.9+/−0.5 *(9.3%)*	1.8+/−0.5 *(9.2%)*
Deaths from either cause	7.2+/−2.6 *(6.8%)*	1.6+/−0.5 *(6.7%)*	2.1+/−0.7 *(6.7%)*	1.3+/−0.4 *(6.9%)*	2.9+/−1.0 *(6.8%)*
Higher risk reduction with each gram salt reduced**					
Incident MI	147.1+/−24.8 *(32.0%)*	61.0+/−10.3 *(31.6%)*	21.5+/−3.6 *(32.1%)*	35.7+/−6.0 *(32.1%)*	62.0+/−10.4 *(32.0%)*
Incident Stroke	9.3+/−1.6 *(44.9%)*	8.8+/−1.6 *(44.4%)*	9.9+/−1.7 *(45.0%)*	9.7+/−1.7 *(45.0%)*	9.5+/−1.7 *(44.9%)*
Deaths from either cause	37.9+/−8.7 *(32.2%)*	8.9+/−2.0 *(33.1%)*	10.4+/−2.3 *(32.9%)*	6.5+/−1.4 *(33.7%)*	15.1+/−3.4 *(33.1%)*

Projected Estimates of Reductions in Cardiovascular Disease from a Dietary Salt Reduction Target of 3 g/day achieved over 30 years (via a linear reduction in intake of 0.1 g/year), in the Main Simulation and According to Various Assumptions about Differential Salt Sensitivity and Blood Pressure Reduction Benefits in the Sensitivity Analyses. MI and stroke incidence includes both new cases and recurrent events.

* Cardiovascular benefit of lowering blood pressure was equivalent to two-thirds of the benefit for a person whose native blood pressure was at that lower blood pressure level [42].

** While the baseline simulation implements the results of a meta-analysis that does not reveal greater salt sensitivity among the elderly [27], we also simulated the case in which each gram reduction in salt intake leads to a greater reduction in blood pressure among older cohorts, as per some clinical trials [31], [32], in which the change in systolic pressure = −0.0598 * (mmol salt reduction)−0.0431 * (age-48)) (see Text S1) [26].

**Figure 2:**
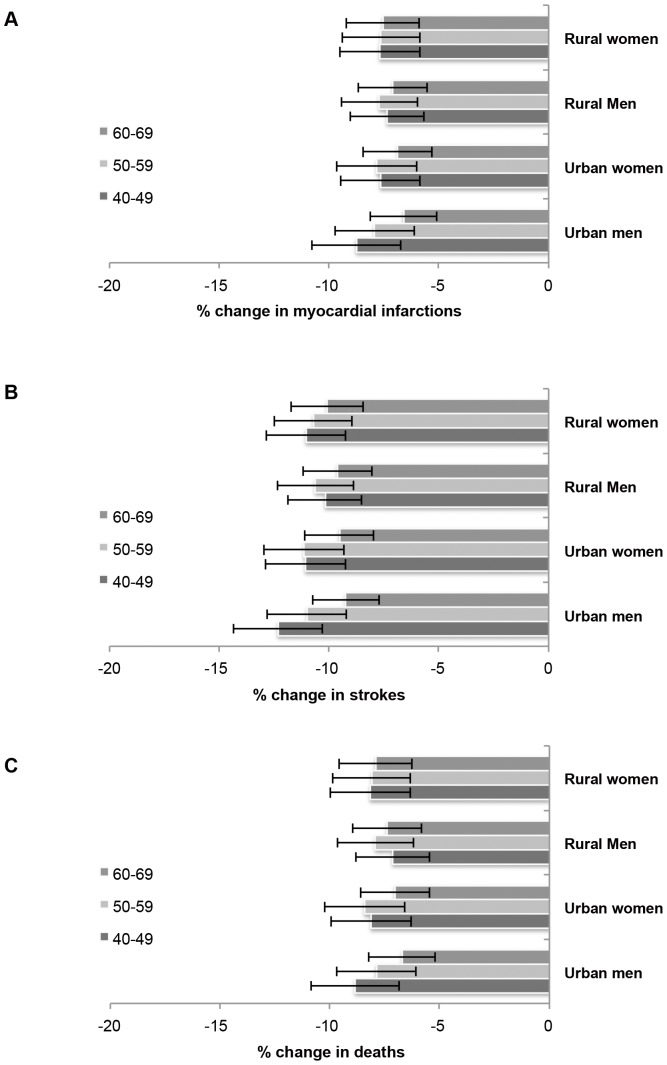
Impact of salt reduction on cardiovascular events and deaths. Projected Reductions in Cardiovascular Events Given a Dietary Salt Reduction Target of 3 g/day over 30 years (via a linear reduction in intake of 0.1 g/year) among Urban Men, Urban Women, Rural Men, and Rural Women, According to Age Cohort. Confidence intervals reflect 2 standard deviations around the mean result from 10,000 simulations. The estimated number of averted cases per year in each cohort (incorporating the population size and rate of events in each cohort) are provided in Table S7. Panel A: Change in new and recurrent MIs. Panel B: Change in new and recurrent strokes. Panel C: Change in deaths from MIs and strokes.

Both sexes and persons in both urban and rural zones were likely to benefit from reductions in salt intake according to our model (Figure 2). However, the anticipated benefits were largest among young urban men. Urban men in the 40 to 49 year old age cohort experienced the greatest reduction in the rate of MI (−8.8%+/−1.4%), stroke (−12.3%+/−2.0%) and overall deaths (−8.8%+/−2.0). However, the percentage decline in MIs, strokes and deaths among all cohorts was within 3% of the changes among this young urban male cohort (Table 2), such that all groups received at least a 6.6% decline in events, and the overall population reduction in MIs was 7.6% (+/−1.3%), in strokes was 10.7% (+/−1.8%) and in deaths was 7.9% (+/−1.7%).

To determine the actual number of averted events in each group, these percentage declines must be weighted by the absolute rate of events and the relative population sizes of each cohort. When accounting for population size projections and the absolute risk of events in each cohort, the largest number of MIs prevented in India would be among urban men in the 50–59 year cohort (96,000 MIs averted per year, +/−16,000); this group would also experience the greatest number of averted deaths (22,000 deaths averted per year, +/−5,000). The greatest number of strokes averted would be among rural men in the 60–69 year age cohort (6,800 strokes averted per year, +/−1,100).

Overall, rural populations accounted for 27% of averted MIs (+/−4.3%), 57% of averted strokes (+/−9.5%), and 32% of averted deaths from MI or stroke (+/−7.6%, Figure 2). Women constituted 37% of averted MIs (+/−6.1%), 53% of averted strokes (+/−8.7%) and 23% of averted deaths (+/−5.4%). More strokes and overall deaths per year were averted among rural than among urban women (4,600+/−750 strokes and 3,000+/−660 deaths among rural women versus 3,100+/−510 strokes and 2,500+/−560 deaths among urban women), while more MIs per year were averted among urban women than among rural women (24,000+/−4,000 MIs among urban women versus 21,000+/−3,300 among rural women). As shown in Figure S4, the number of MIs and strokes averted per year increased among all populations with every additional year of the simulation, suggesting that benefits accrue over a long time course.

### Iodine deficiency

Under a highly-pessimistic set of assumptions (Box S1), a reduction in dietary salt intake of 3 grams would be anticipated to produce at most 1,600 new cases of iodine deficiency (defined as any low level of blood iodine) that would otherwise be averted (95% CI: 660–2,300) which would correspond to a <0.0001% rise in iodine deficiency based on current estimates from India [30]. The worst-affected province would be Andra Pradesh, experiencing 95% of those excess cases (1500; 95% CI: 650–2,200) because more than any other state, a substantive portion of the population receives salt iodized at low levels of iodine per gram of salt (Figure S3), such that the dietary salt reduction program could reduce overall iodine intake from just above required levels to just below required levels among some populations in that province.

### Sensitivity Analyses

If lowering hypertensive blood pressures through reduced salt intake is not as beneficial as having always maintained a lower blood pressure (see Box S1 for relative risk change), the expected health benefits of salt reduction would be decreased by approximately 15% (Table 2). If salt reduction produced greater blood pressure reduction for each gram salt reduced (also a relative risk modification, Box S1), as suggested by some clinical trials showing greater salt sensitivity among older persons than younger people [31], [32], the predicted systolic blood pressure changes would be about four times that of the baseline meta-analysis results (e.g., a 55 year old urban man would be predicted to experience a 0.8 mmHg decline in systolic pressure in the baseline scenario for each 1gram salt reduction, but a 3.3 mmHg decline in this optimistic scenario), and the benefits of salt reduction in the model correspondingly would increase by approximately a factor of four over the baseline simulation (Table 2). Additional sensitivity analyses involving only a 1 g/day salt reduction, or as much as a 4 g/day salt reduction over 30 years were also performed, and showed essentially linear changes to MI and stroke deaths (Tables S8 and S9 and Figures S5 and S6).

## Discussion

Using a mathematical model of dietary salt intake, blood pressure and subsequent MI and stroke events in India, we found that dietary salt reduction of 3 grams over 30 years would be anticipated to avert nearly 400,000 cases and about 81,000 deaths from MI and stroke in India. These reductions correspond to between 6.6% and 12.3% reductions in the rate of MIs and strokes among different subpopulations of the country, averting between 6.7% and 8.8% of MI and stroke-related deaths. Males, and urban men of working age in particular, would experience the greatest predicted benefits from dietary salt reduction. However, the benefits would accrue across both men and women, in both urban and rural populations. Over half of strokes, for example, would be averted among women, and one out of every three deaths averted would be among rural populations. Only under highly pessimistic assumptions would salt reduction at these levels increase risks of iodine deficiency by <0.001% percentage point, corresponding to about 200 new cases of iodine deficiency (95% CI: 120–273).

Before analyzing the policy implications of these findings, we must note several important limitations. First, we simulated the effects of salt reduction on blood pressure using the most conservative meta-analysis results available to date. These studies mostly derive from Western nations, but Intersalt and related studies suggest that the relationships between salt and blood pressure, and between blood pressure and cardiovascular disease are universal among humans [19], [27], [33]. However, there have been some studies suggesting that individuals in South Asian communities might be genetically predisposed to hypertension and various cardiovascular diseases [34], [35]. Although salt intake will likely benefit these individuals, it remains unclear if these persons will see a similar reduction in hypertension and the associated risks as the rest of the population; this is a limitation of our predictions. Second, we assumed that the impact of salt reduction on cardiovascular disease was linear and entirely mediated by the relationship between salt and blood pressure, although some experts have argued that additional mechanisms may contribute to further mortality benefits [36] and that the salt-hypertension relationship may be non-linear [31], [37] in a direction that would render our results conservative. Additionally, in the UK, a 0.9 g/day reduction in salt intake was achieved over 4 years (∼0.225 g/day per capita per year) [12], [38]—more than twice as large as our modeling approach based on the Finnish North Karelia experience, further suggesting that our baseline model scenario may be conservative. Fourth, we focused on the 40 to 69 year old groups given the higher reliability of data available for this age range from India, the rise of hypertension among young males may indicate benefit in other age cohorts [2]. Fifth, we did not explicitly simulate migration but rather inferred migratory effects using Indian Census data; this may limit the applicability of our model in future years when migration patterns may change. Finally, we did not assess ages beyond 40 to 69 years old, or additional diseases related to hypertension such as renal disease or gastric cancer.

Our model produced estimates of benefit consistent with models of salt reduction in Western countries [25], [26], suggesting robust findings among independent researchers and alternative models. Our model contributes significant new information in that it directly incorporates new data from a key developing country to investigate whether salt reduction would accrue benefits outside of urban populations eating Western diets, and whether perverse outcomes are likely to occur. We included time-trends in MI/stroke risk as well as in mortality rates from other diseases (Table S2), to account for the rapid change in disease risk over time as infectious disease rates decline and chronic disease rates rise. Our results are highly relevant to other countries, as similar rates of salt intake and associated hypertension have been reported in China, Colombia and Mexico [39]. Given that hypertension has now become the leading modifiable risk factor for death in the world [40], India's path to controlling salt intake may define a paradigm for rapidly-developing countries.

However, the projected benefits in our model were smaller than one older analysis of salt intake [41], in part because we relied on a more recent meta-analysis [27] estimating a more limited benefit of salt reduction (the older analysis used more optimistic studies conducted prior to 1991) [42]. The older study also lacked population-representative hypertension data, requiring the authors to extrapolate data from a small single-locale survey to the entire South Asian region; we were fortunate to access the WHO SAGE study to reflect population-representative levels of blood pressure and associated risk in both urban and rural regions, stratified by age and gender [43].

Potential unintended consequences, like iodine deficiency, were found to be very small, even under a worst-case scenario assuming extremely high iodine requirements and low levels of iodine intake. Most iodine deficiency in India occurs in areas without adequate access to iodized salt (not due to inadequate intake of iodized salt); reductions in salt intake would therefore not affect the likelihood of iodine deficiency in such areas [44]. In some regions, however, salt is inadequately iodized, such that dietary salt reduction could induce new iodine deficiency. However, we found that because of the minimal amount of iodine required to meet nutritional needs, dietary salt reduction programs are unlikely to produce substantial new iodine deficiency in such regions, consistent with a recent World Health Organization review [45], [46].

How to optimally implement salt reduction initiatives in India remains unclear. Regulatory interventions such as substituting potassium for sodium or reducing “hidden” sodium in processed foods may be more effective than educational interventions [12], [17], [47]. While concerns have been raised over the economic impact of regulatory approaches, experience in the UK resulted in population-wide reduction in dietary salt intake without reduction in food product sales, problems with food preservation or consumer complaints about food taste [38]. Further work is being initiated to gather data to study the cost-effectiveness of alternative salt reduction approaches [29], [39].

Overall, our findings indicate that moderate reductions in dietary salt intake in India, similar to salt reductions achieved in Western populations [10], could substantially reduce cardiovascular morbidity and mortality in both genders, among both middle-aged and older age groups, and among both urban and rural populations in the country.

## Supporting Information

Box S1 **Additional modeling scenarios.**
(DOC)

Figure S1 **Demographic projections for India by age, gender and location.**
(TIFF)

Figure S2 **Face validity of the model compared to independent projections for (A) MI and (B) stroke.**
(TIF)

Figure S3 **Iodine content per gram of salt among Indian provinces.**
(TIFF)

Figure S4 **Number of incident and recurrent MIs, strokes and associated deaths averted over time Given a Dietary Salt Reduction Target of 3 g/day achieved over 30 years.**
(TIFF)

Figure S5 **Projected Reductions in Cardiovascular Events Given a Dietary Salt Reduction Target of 1 g/day achieved over 30 years.** (A) MI, (B) stroke, (C) associated deaths.
(TIF)

Figure S6 **Projected Reductions in Cardiovascular Events Given a Dietary Salt Reduction Target of 4 g/day achieved over 30 years.** (A) MI, (B) stroke, (C) associated deaths.
(TIF)

Table S1 **Risk of MI and stroke by age, gender and location.**
(DOC)

Table S2 **Time trends in MI and stroke risk by age, gender and location.**
(DOC)

Table S3 **Case fatality rates for MI and stroke by age, gender and location.**
(DOC)

Table S4 **Mortality rates from non-MI and non-stroke causes by age, gender and location.**
(DOC)

Table S5 **Time trends in non-MI and non-stroke mortality by age, gender and location.**
(DOC)

Table S6 **Baseline hypertension prevalence by age, gender and location.**
(DOC)

Table S7 **Annual number of averted MIs, strokes and associated deaths by age, gender and location given a dietary salt reduction target of 3 g/day achieved over 30 years.**
(DOC)

Table S8 **Annual number of averted MIs, strokes and associated deaths by age, gender and location given a dietary salt reduction target of 1 g/day achieved over 30 years.**
(DOC)

Table S9 **Annual number of averted MIs, strokes and associated deaths by age, gender and location given a dietary salt reduction target of 4 g/day achieved over 30 years.**
(DOC)

Text S1 **Description of data sources and modeling approach.**
(DOC)
