# Supplementary material for: Dietary Salt Reduction and Cardiovascular Disease Rates in India: A Mathematical Model
Source: PLoS One. 2012 Sep 6;7(9):e44037. doi: 10.1371/journal.pone.0044037 (PMC3435319; doi:10.1371/journal.pone.0044037)
Supplement: Box S1 — Additional modeling scenarios. (DOC) [file pone.0044037.s001.doc]

**Box S1. Alternative modeling scenarios**

*Degree of salt reduction*

Our baseline simulation drew upon the Finnish North Karelia experience, where salt intake was reduced from 12 g/day to 9 g/day over 30 years (by some accounts, the amount of salt reduction may have been higher, on the order of 5g , but we chose the most conservative estimate). A 3 gram reduction over 30 years is thought to also be feasible for developing countries including India according to recent assessments of current levels of dietary salt intake in India and potential interventions . Hence, in the baseline scenario, we considered a 0.1 g/year reduction over 30 years (a total of 3 g between the years 2013 and 2043). In sensitivity analyses, we examined the impact of varying the degree of salt reduction between 1 g/d and 4 g/d of total salt reduction over the 30 year time period.

*Impact of blood pressure reduction on outcomes*

We conducted sensitivity analyses to compare alternative estimates of the impact of salt reduction on blood pressure reduction. In our baseline model, we use the most conservative estimates from a recent meta-analysis , and did not assume greater salt sensitivity among older groups. In a more optimistic scenario, based on clinical trials of salt reduction, we simulated greater blood pressure lowering among older adults (see Table 2 for parameters and results) . Conversely, we also performed sensitivity analyses to simulate the more pessimistic case suggested by some studies that lowering high blood pressure through salt reduction does not confer as much benefit as having maintained a low blood pressure (i.e., avoiding hypertension altogether may confer more benefits than reducing hypertension; see Table 2 for parameters and results) .

*Uncertainty analyses*

Monte Carlo simulations were performed to estimate the uncertainty around model projections by sampling from the probability distributions of the input parameters. In Supporting Information Tables 1 through 5, we list the mean and 95% confidence intervals around all of the transition probabilities employed in the model. For each simulation, we sampled 10,000 times from Gaussian distributions defined by these means and intervals, and report the mean and 95% confidence intervals around each of our results.

*Iodine deficiency*

We explored whether reductions in dietary salt intake were likely to increase iodine deficiency in India. Currently, analyses of iodine deficiency suggest that goiter and related iodine deficiency disorders occur in Indian regions where salt is typically non-iodized; therefore, dietary salt reduction would not affect the likelihood of iodine deficiency in such regions . Some populations are, however, at risk for iodine deficiency in the context of having access to salt iodized at low levels of iodine per each gram of salt. We calculated the probability that any individual of any age could experience inadequate iodine intake because of a dietary salt reduction program. The leading international council for iodine deficiency has stated that “a particle [of iodine] the size of a pinhead is sufficient for one person for one month” , though we simulated a higher requirement to produce conservative projections. To provide a highly pessimistic estimate of iodine deficiency, we assumed that an individual would require 200 micrograms of iodine per day (a high-end estimate ), and that individuals would not have any other means of iodine intake besides salt. We used the lowest quantity of per capita salt consumption observed in recent surveys (4.9 grams of salt per person per day ) to produce a pessimistic estimate of iodine intake from salt. The Indian National Family Health Survey provides estimates of iodine content per gram of salt (Supporting Information Figure S3) from household survey data in each province , which we multiplied by the estimate of salt intake per person per day to obtain an estimate of typical iodine intake per person per day in each province. The probability of iodine deficiency in each province was then calculated from a standard Poisson cumulative distribution function around this typical iodine intake level (the probability of deficiency is the value of the distribution function at the 200 microgram threshold, ). We repeated the calculation after simulating a 3 gram per day salt intake reduction (to 1.9 grams/person/day) to estimate the number of Indians who would become newly iodine deficient as a result of dietary salt reduction.
