# Supplementary figures and images for: Dietary Salt Reduction and Cardiovascular Disease Rates in India: A Mathematical Model
Source: PLoS One. 2012 Sep 6;7(9):e44037. doi: 10.1371/journal.pone.0044037 (PMC3435319; doi:10.1371/journal.pone.0044037)

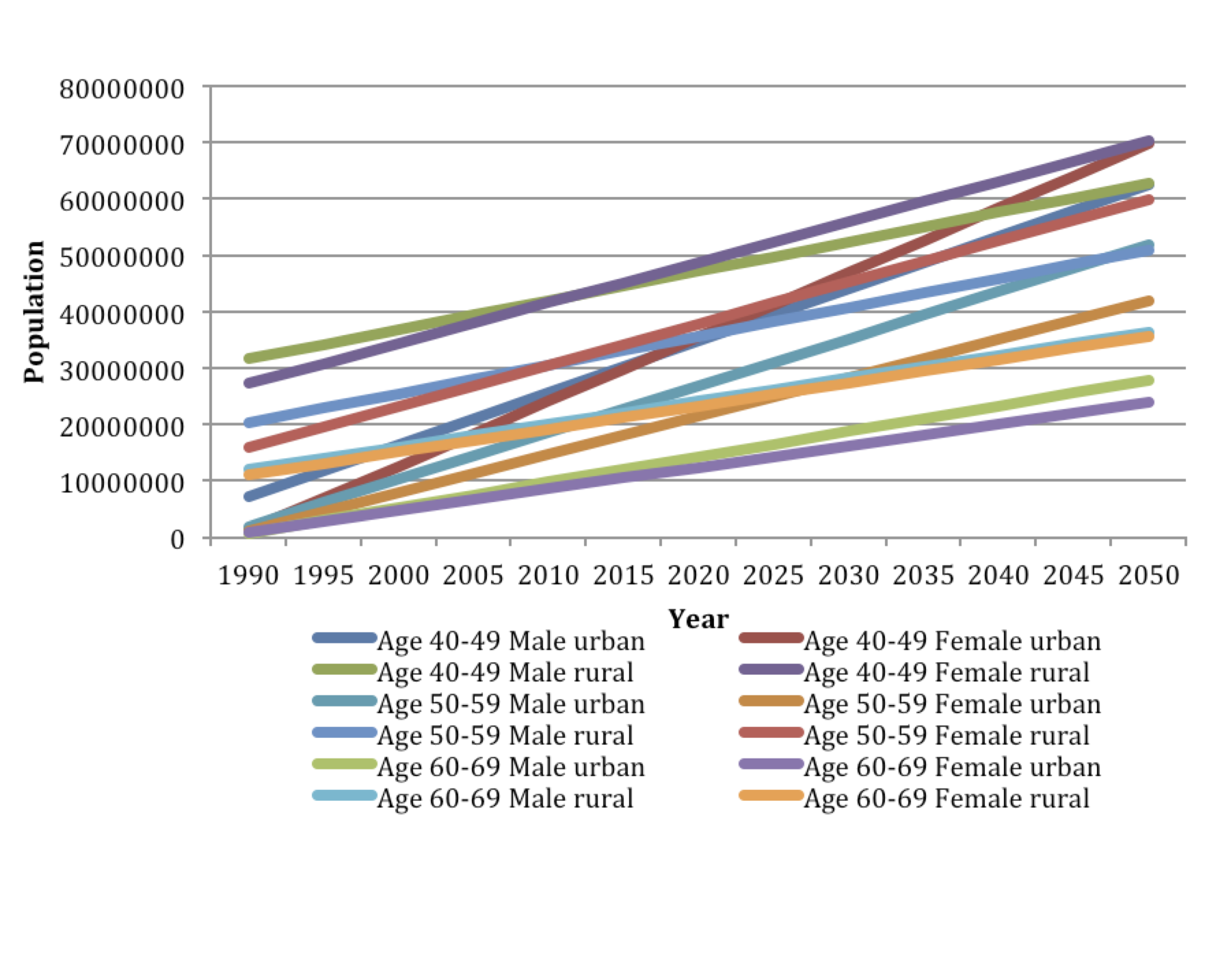

Supplement: Figure S1 — Demographic projections for India by age, gender and location. (TIFF) [file pone.0044037.s002.tiff]

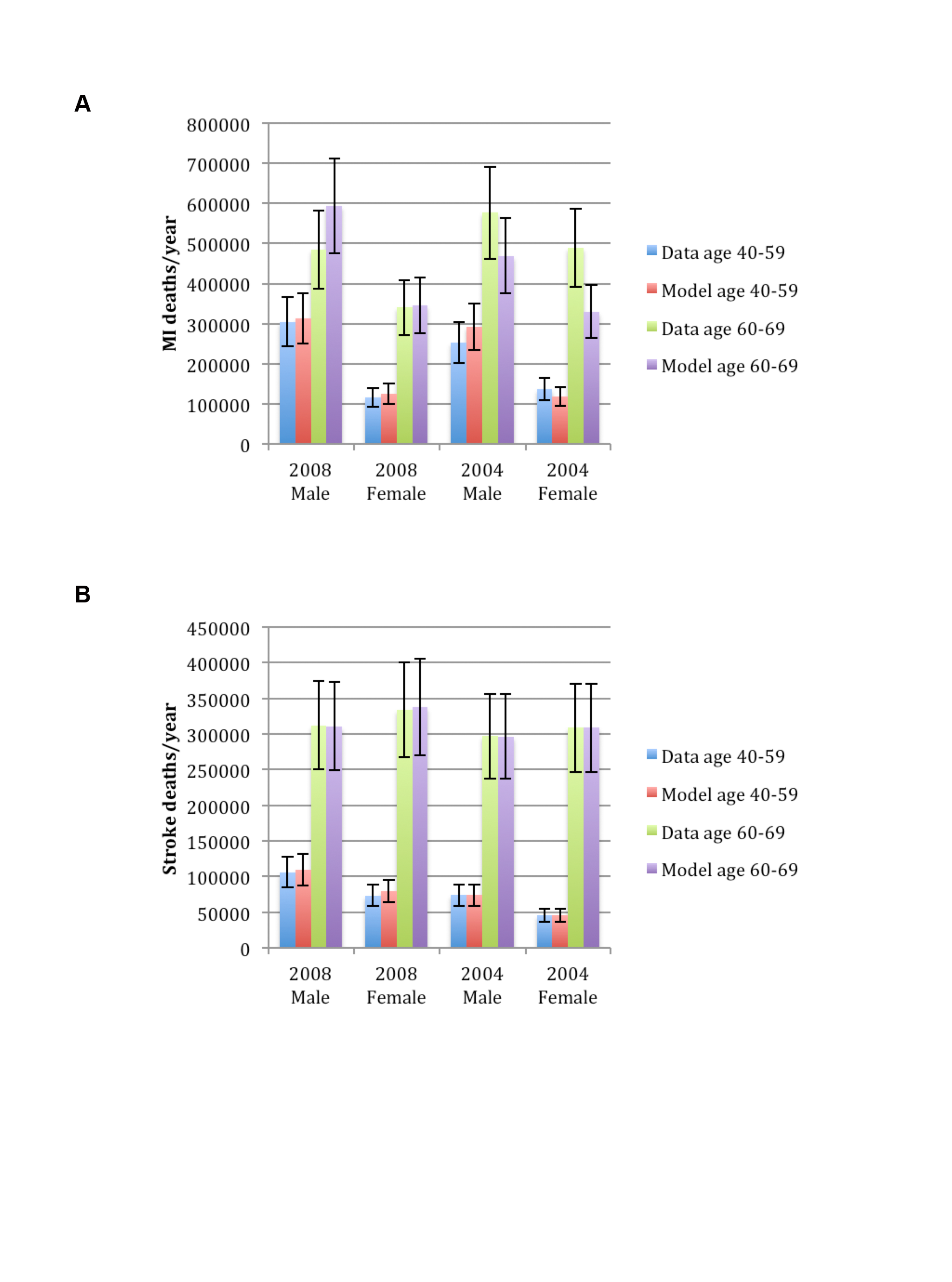

Supplement: Figure S2 — Face validity of the model compared to independent projections for (A) MI and (B) stroke. (TIF) [file pone.0044037.s003.tif]

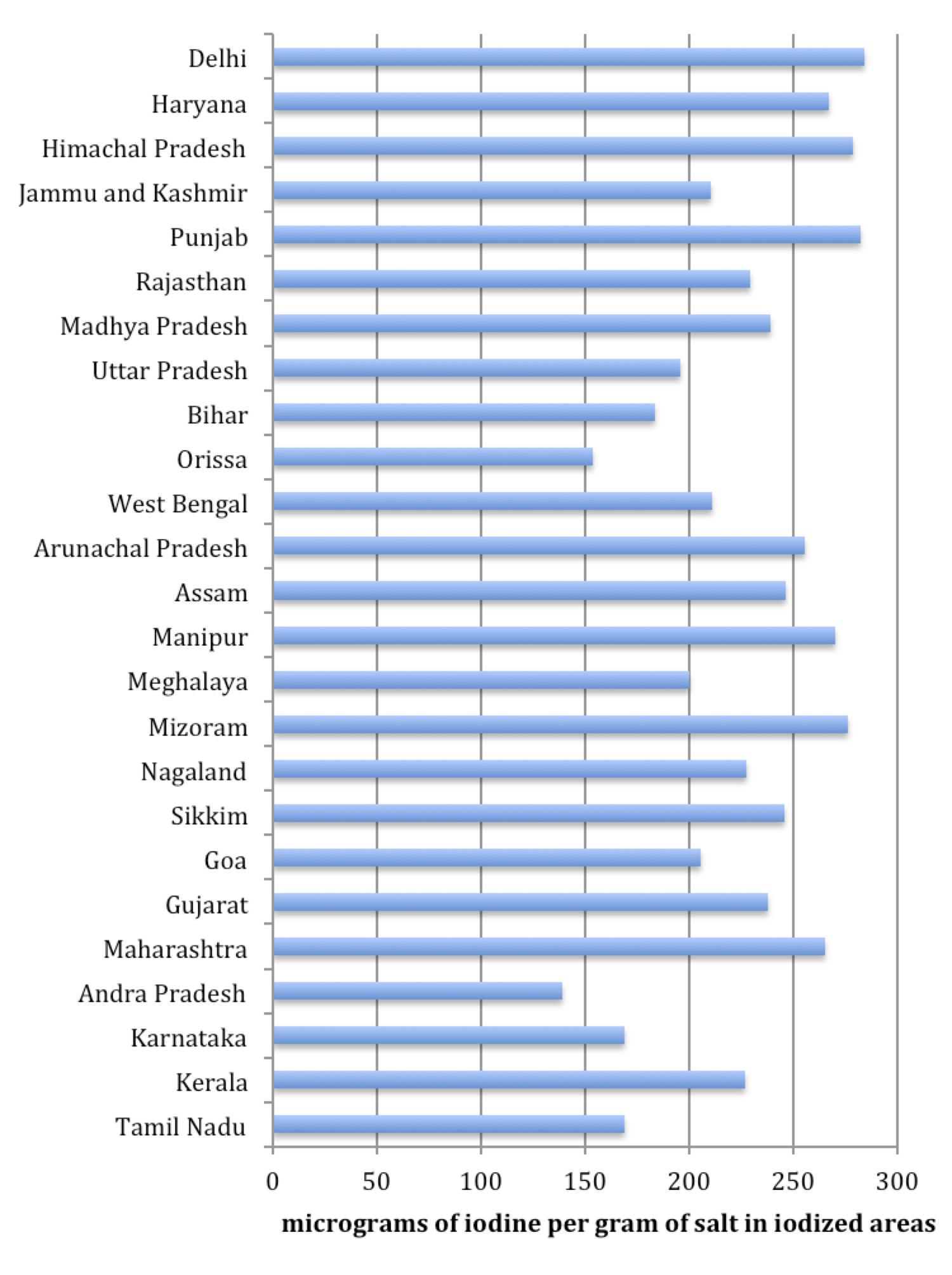

Supplement: Figure S3 — Iodine content per gram of salt among Indian provinces. (TIFF) [file pone.0044037.s004.tiff]

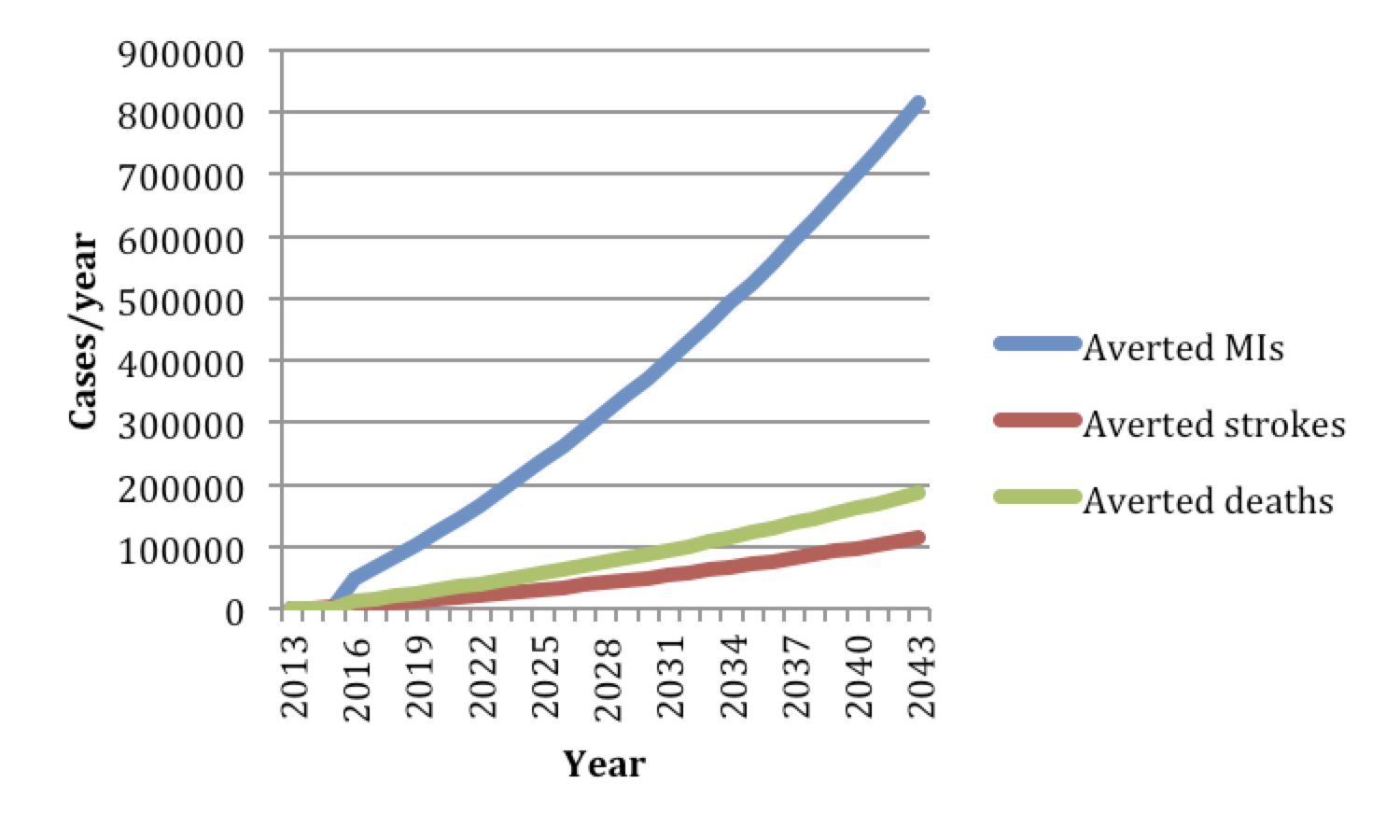

Supplement: Figure S4 — Number of incident and recurrent MIs, strokes and associated deaths averted over time Given a Dietary Salt Reduction Target of 3 g/day achieved over 30 years. (TIFF) [file pone.0044037.s005.tiff]

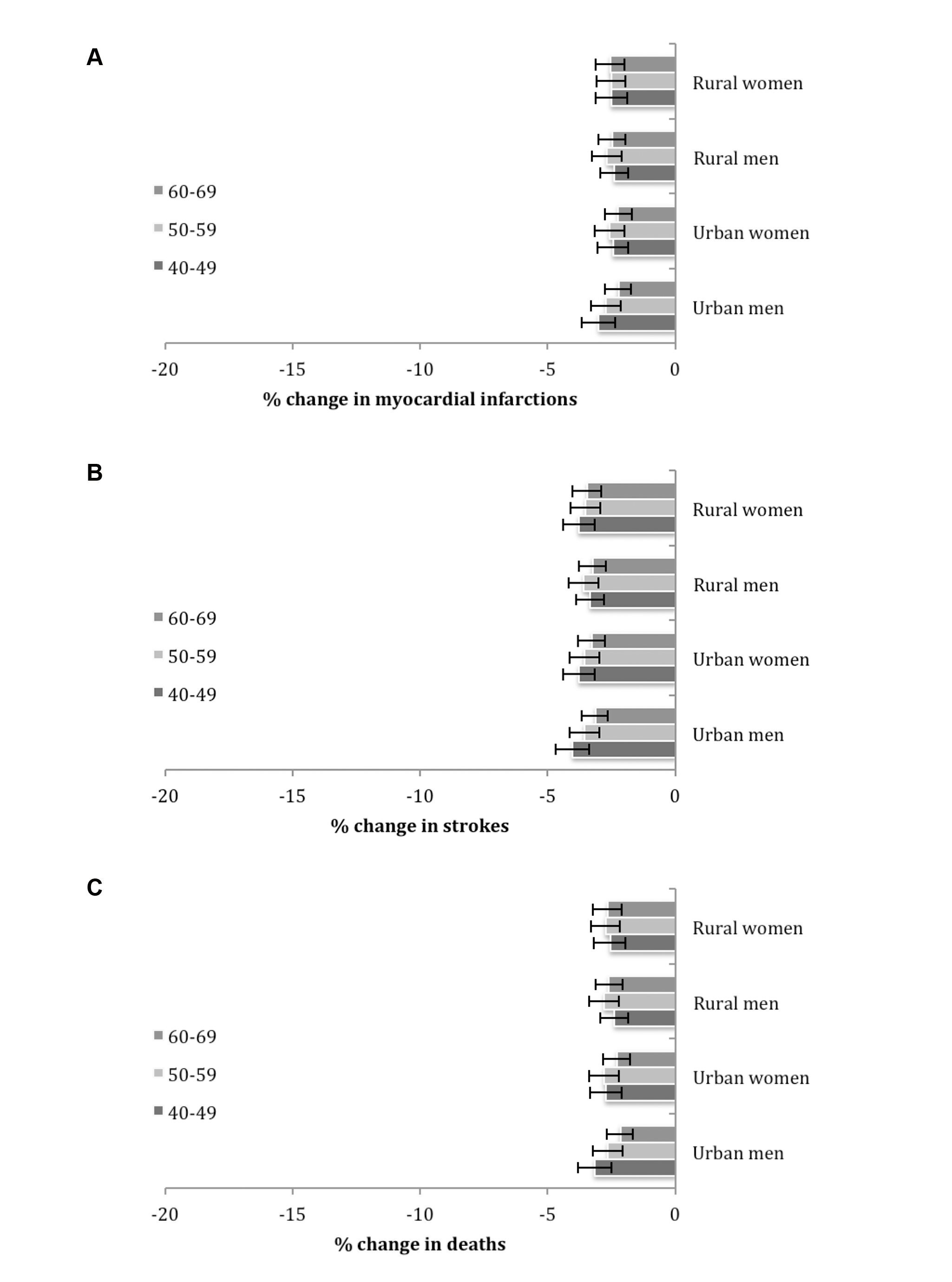

Supplement: Figure S5 — Projected Reductions in Cardiovascular Events Given a Dietary Salt Reduction Target of 1 g/day achieved over 30 years. (A) MI, (B) stroke, (C) associated deaths. (TIF) [file pone.0044037.s006.tif]

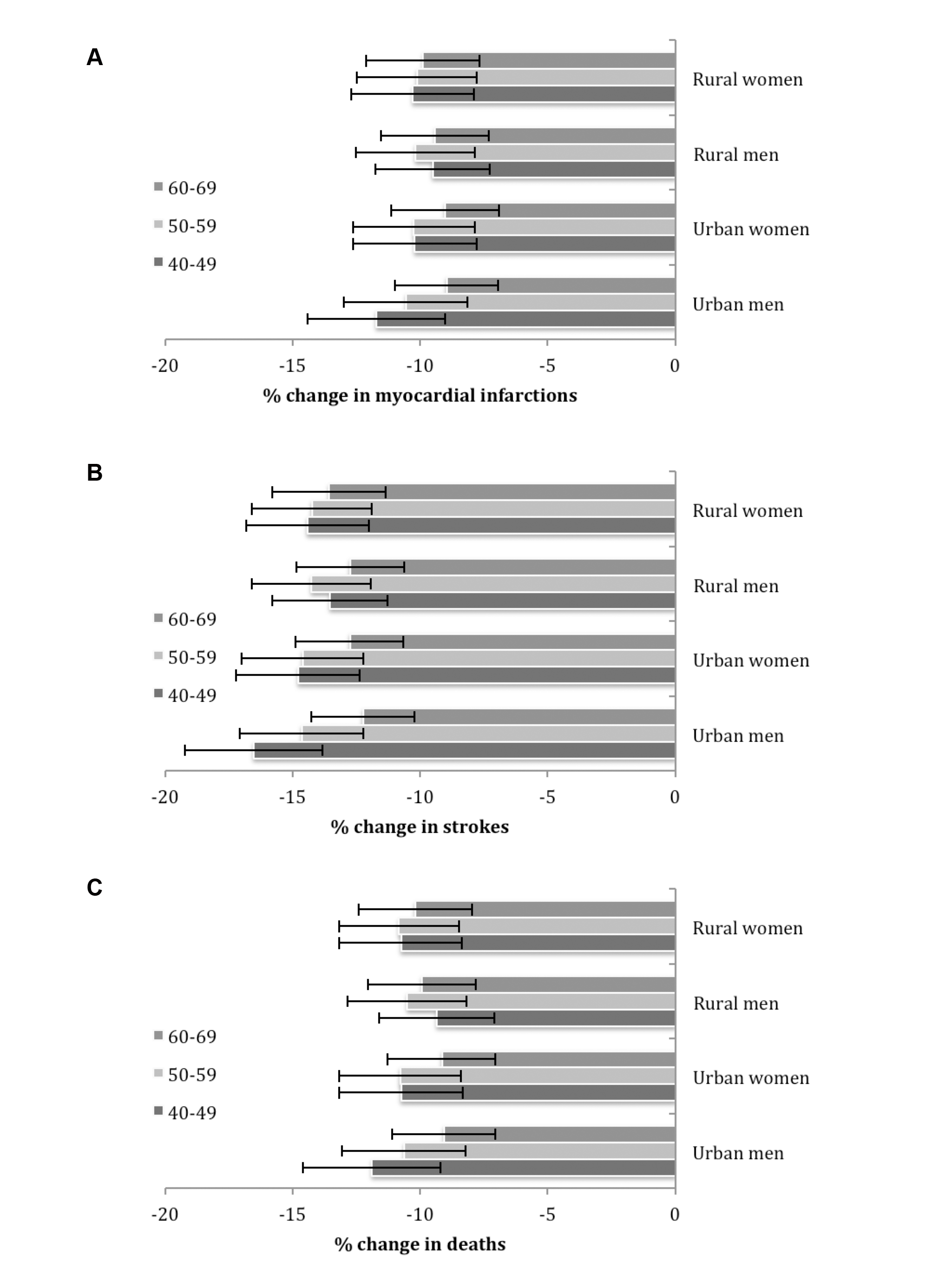

Supplement: Figure S6 — Projected Reductions in Cardiovascular Events Given a Dietary Salt Reduction Target of 4 g/day achieved over 30 years. (A) MI, (B) stroke, (C) associated deaths. (TIF) [file pone.0044037.s007.tif]
