# Supplementary material for: Dietary Salt Reduction and Cardiovascular Disease Rates in India: A Mathematical Model
Source: PLoS One. 2012 Sep 6;7(9):e44037. doi: 10.1371/journal.pone.0044037 (PMC3435319; doi:10.1371/journal.pone.0044037)
Supplement: Table S1 — Risk of MI and stroke by age, gender and location. (DOC) [file pone.0044037.s008.doc]

**SI Table S1. Risk of MI and stroke by age, gender and location.** Risk displayed is annual risk per 1,000 persons for the year 1998 , from which the time-trend in SI Table 2 is applied for future years to produce projections from 1998 through 2050. 95% confidence intervals are listed in parentheses. Recurrence rates are 250.8 per 1,000 (95% CI: 130.66-459.0) for MI and 317.1 per 1,000 (95% CI: 220.1-500.0) for stroke, which applies to the population having a prior history of MI or stroke; the population with a prior history of either condition in the year 1998 was obtained from the WHO to initiate the simulation.

1. Incident MI rate per 1,000 persons.

| Age | Male urban | Female urban | Male rural | Female rural |
| --- | --- | --- | --- | --- |
| 40-49 | 19.3 (16.2-22.4) | 14.3 (7.9-20.6) | 5.2 (5.1-5.3) | 9.5 (7.3-11.8) |
| 50-59 | 51.2 (43.3-59.1) | 20.1 (7.4-32.8) | 6.5 (4.9-8.1) | 14.6 (12.4-16.7) |
| 60-69 | 86.3 (82.3-90.2) | 87.7 (79.5-95.9) | 6.6 (4.7-8.5) | 29.4 (25.9-32.9) |

1. Incident stroke rate per 1,000 persons.

| Age | Male urban | Female urban | Male rural | Female rural |
| --- | --- | --- | --- | --- |
| 40-49 | 0.21 (0.17-0.25) | 0.69 (0.54-0.84) | 0.21 (0.17-0.25) | 0.69 (0.54-0.84) |
| 50-59 | 2.2 (1.1-3.3) | 2.0 (1.2-2.8) | 2.2 (1.1-3.3) | 2.0 (1.2-2.8) |
| 60-69 | 4.7 (4.6-4.8) | 3.4 (2.9-3.8) | 4.7 (4.6-4.8) | 3.4 (2.9-3.8) |
