# Supplementary material for: Dietary Salt Reduction and Cardiovascular Disease Rates in India: A Mathematical Model
Source: PLoS One. 2012 Sep 6;7(9):e44037. doi: 10.1371/journal.pone.0044037 (PMC3435319; doi:10.1371/journal.pone.0044037)
Supplement: Table S2 — Time trends in MI and stroke risk by age, gender and location. (DOC) [file pone.0044037.s009.doc]

**SI Table S2. Time trends in MI and stroke rates by age, gender and location .**

1. Change in MI incidence per 1,000 persons per year.

| Age | Male urban | Female urban | Male rural | Female rural |
| --- | --- | --- | --- | --- |
| 40-49 | 0.020 | -0.024 | 0.020 | -0.024 |
| 50-59 | 0.020 | -0.024 | 0.020 | -0.024 |
| 60-69 | -0.65 | -0.97 | -0.65 | -0.97 |

1. Change in stroke incidence per 1,000 persons per year.

| Age | Male urban | Female urban | Male rural | Female rural |
| --- | --- | --- | --- | --- |
| 40-49 | 0.017 | 0.017 | 0.017 | 0.017 |
| 50-59 | 0.017 | 0.017 | 0.017 | 0.017 |
| 60-69 | 0.045 | 0.045 | 0.045 | 0.045 |
