# Supplementary material for: Dietary Salt Reduction and Cardiovascular Disease Rates in India: A Mathematical Model
Source: PLoS One. 2012 Sep 6;7(9):e44037. doi: 10.1371/journal.pone.0044037 (PMC3435319; doi:10.1371/journal.pone.0044037)
Supplement: Table S3 — Case fatality rates for MI and stroke by age, gender and location. (DOC) [file pone.0044037.s010.doc]

**SI Table S3. Case fatality rates for MI and stroke by age, gender and location .**

1. MI case fatality rate (%)

| Age | Male urban | Female urban | Male rural | Female rural |
| --- | --- | --- | --- | --- |
| 40-49 | 11.4 (9.9-13.0) | 3.3 (2.8-3.8) | 18.6 (13.8-23.5) | 4.7 (4.2-5.3) |
| 50-59 | 22.1 (19.7-24.5) | 8.1 (6.4-9.7) | 52.9 (43.7-62.1) | 10.9 (8.5-13.3) |
| 60-69 | 45.6 (32.2-59.0) | 25.2 (15.9-34.6) | 81.6 (71.3-91.8) | 34.9 (22.5-47.3) |

1. Stroke case fatality rate (%)

| Age | Male urban | Female urban | Male rural | Female rural |
| --- | --- | --- | --- | --- |
| 40-49 | 5.6 (4.1-7.1) | 5.8 (4.4-7.2) | 5.6 (4.1-7.1) | 5.8 (4.4-7.2) |
| 50-59 | 9.8 (8.8-10.7) | 9.8 (8.8-10.7) | 9.8 (8.8-10.7) | 9.8 (8.8-10.7) |
| 60-69 | 14.2 (13.5-15.0) | 14.4 (13.6-15.2) | 14.2 (13.5-15.0) | 14.4 (13.6-15.2) |
