# Supplementary material for: Dietary Salt Reduction and Cardiovascular Disease Rates in India: A Mathematical Model
Source: PLoS One. 2012 Sep 6;7(9):e44037. doi: 10.1371/journal.pone.0044037 (PMC3435319; doi:10.1371/journal.pone.0044037)
Supplement: Table S4 — Mortality rates from non-MI and non-stroke causes by age, gender and location. (DOC) [file pone.0044037.s011.doc]

**SI Table S4. Mortality rates from non-MI and non-stroke causes by age, gender and location .** Rates are displayed per 1,000 people for the year 1998, after which the time trend displayed in SI Table 5 is employed to predict future rates.

| Age | Male urban | Female urban | Male rural | Female rural |
| --- | --- | --- | --- | --- |
| 40-49 | 6.0 (4.8-7.2) | 5.3 (4.2-6.4) | 6.0 (4.8-7.2) | 5.3 (4.2-6.4) |
| 50-59 | 16.4 (13.1-19.7) | 14.6 (11.6-17.5) | 16.4 (13.1-19.7) | 14.6 (11.6-17.5) |
| 60-69 | 26.8 (21.5-32.2) | 23.8 (19.0-28.6) | 26.8 (21.5-32.2) | 23.8 (19.0-28.6) |
