# Supplementary material for: Dietary Salt Reduction and Cardiovascular Disease Rates in India: A Mathematical Model
Source: PLoS One. 2012 Sep 6;7(9):e44037. doi: 10.1371/journal.pone.0044037 (PMC3435319; doi:10.1371/journal.pone.0044037)
Supplement: Table S5 — Time trends in non-MI and non-stroke mortality by age, gender and location. (DOC) [file pone.0044037.s012.doc]

**SI Table S5. Time trends in non-MI and non-stroke mortality by age, gender and location (per 1,000 persons per year) .**

| Age | Male urban | Female urban | Male rural | Female rural |
| --- | --- | --- | --- | --- |
| 40-49 | -0.011 | -0.013 | -0.011 | -0.013 |
| 50-59 | 0.032 | 0.023 | 0.032 | 0.023 |
| 60-69 | 0.074 | 0.060 | 0.074 | 0.060 |
