# Supplementary material for: Dietary Salt Reduction and Cardiovascular Disease Rates in India: A Mathematical Model
Source: PLoS One. 2012 Sep 6;7(9):e44037. doi: 10.1371/journal.pone.0044037 (PMC3435319; doi:10.1371/journal.pone.0044037)
Supplement: Table S6 — Baseline hypertension prevalence by age, gender and location. (DOC) [file pone.0044037.s013.doc]

**SI Table S6. Hypertension prevalence by age, gender and location .**

| Age | Urban men | Urban women | Rural men | Rural women |
| --- | --- | --- | --- | --- |
| 40-49 | 0.454 | 0.247 | 0.206 | 0.221 |
| 50-59 | 0.343 | 0.297 | 0.311 | 0.256 |
| 60-69 | 0.297 | 0.257 | 0.322 | 0.343 |
