# Supplementary material for: Dietary Salt Reduction and Cardiovascular Disease Rates in India: A Mathematical Model
Source: PLoS One. 2012 Sep 6;7(9):e44037. doi: 10.1371/journal.pone.0044037 (PMC3435319; doi:10.1371/journal.pone.0044037)
Supplement: Table S7 — Annual number of averted MIs, strokes and associated deaths by age, gender and location given a dietary salt reduction target of 3 g/day achieved over 30 years. (DOC) [file pone.0044037.s014.doc]

**SI Table S7. Annual number of averted MIs, strokes and associated deaths by age, gender and location given a dietary salt reduction target of 3g/day achieved over 30 years.** 95% confidence intervals are displayed in parentheses. Results are presented to a precision of two significant digits.

(A) Averted MIs per year

| Age | Urban men | Urban women | Rural men | Rural women |
| --- | --- | --- | --- | --- |
| 40-49 | 49000 (41000-57000) | 25000 (21000-29000) | 13000 (11000-15000) | 19000 (16000-23000) |
| 50-59 | 96000 (80000-110000) | 22000 (18000-25000) | 13000 (11000-16000) | 24000 (20000-28000) |
| 60-69 | 38000 (32000-44000) | 27000 (23000-32000) | 4700 (3900-5400) | 17000 (14000-20000) |

(B) Averted strokes per year

| Age | Urban men | Urban women | Rural men | Rural women |
| --- | --- | --- | --- | --- |
| 40-49 | 700 (590-810) | 2500 (2000-2900) | 670 (560-780) | 2800 (2400­-3400) |
| 50-59 | 5300 (4400-6200) | 4300 (3600-5000) | 5700 (4800-6700) | 6400 (5400-7400) |
| 60-69 | 4500 (3800-5300) | 3000 (2500-3500) | 6800 (5700-7900) | 5200 (4300-6000) |

(C) Averted deaths per year

| Age | Urban men | Urban women | Rural men | Rural women |
| --- | --- | --- | --- | --- |
| 40-49 | 5700 (4400-7000) | 970 (770-1200) | 2300 (1700-2800) | 1100 (870-1300) |
| 50-59 | 22000 (16000-26000) | 2200 (1700-2700) | 7700 (6000-9400) | 3300 (2600-3900) |
| 60-69 | 18000 (14000-22000) | 7400 (5700-9000) | 4600 (3700-5500) | 3100 (5400-8400) |
