# Supplementary material for: Dietary Salt Reduction and Cardiovascular Disease Rates in India: A Mathematical Model
Source: PLoS One. 2012 Sep 6;7(9):e44037. doi: 10.1371/journal.pone.0044037 (PMC3435319; doi:10.1371/journal.pone.0044037)
Supplement: Table S8 — Annual number of averted MIs, strokes and associated deaths by age, gender and location given a dietary salt reduction target of 1 g/day achieved over 30 years. (DOC) [file pone.0044037.s015.doc]

**SI Table S8. Annual number of averted MIs, strokes and associated deaths by age, gender and location given a dietary salt reduction target of 1g/day achieved over 30 years.** 95% confidence intervals are displayed in parentheses. Results are presented to a precision of two significant digits.

(A) Averted MIs per year

| Age | Urban men | Urban women | Rural men | Rural women |
| --- | --- | --- | --- | --- |
| 40-49 | 17000 (14000-20000) | 7600 (6600-8800) | 4200 (3500-4800) | 6200 (5200-7200) |
| 50-59 | 34000 (28000-39000)) | 7100 (6000-8300) | 4800 (4000-5500) | 7900 (6600-9200) |
| 60-69 | 13000 (11000-15000) | 8700 (7300-10000) | 3800 (1400-2000) | 6700 (4900-6700) |

(B) Averted strokes per year

| Age | Urban men | Urban women | Rural men | Rural women |
| --- | --- | --- | --- | --- |
| 40-49 | 220 (180-260) | 860 (720-1000) | 220 (180-250) | 1000 (840-1200) |
| 50-59 | 1700 (1400-2000) | 1300 (1100-1500) | 2000 (1600-2300) | 2100 (1700-2400) |
| 60-69 | 1500 (1300-1800) | 1100 (880-1200) | 2300 (1900-2700) | 1800 (1500-2100 |

(C) Averted deaths per year

| Age | Urban men | Urban women | Rural men | Rural women |
| --- | --- | --- | --- | --- |
| 40-49 | 2100 (1600-2600) | 330 (260-400) | 760 (590-940) | 320 (260-390) |
| 50-59 | 7200 (5500-8800) | 730 (580-880) | 2800 (2200-3400) | 1100 (880-1300) |
| 60-69 | 5600 (4300-6800) | 2300 (1800-2800) | 1700 (1400-2000) | 2300 (1800-2800) |
