# Supplementary material for: Dietary Salt Reduction and Cardiovascular Disease Rates in India: A Mathematical Model
Source: PLoS One. 2012 Sep 6;7(9):e44037. doi: 10.1371/journal.pone.0044037 (PMC3435319; doi:10.1371/journal.pone.0044037)
Supplement: Table S9 — Annual number of averted MIs, strokes and associated deaths by age, gender and location given a dietary salt reduction target of 4 g/day achieved over 30 years. (DOC) [file pone.0044037.s016.doc]

**SI Table S9. Annual number of averted MIs, strokes and associated deaths by age, gender and location given a dietary salt reduction target of 4g/day achieved over 30 years.** 95% confidence intervals are displayed in parentheses. Results are presented to a precision of two significant digits.

(A) Averted MIs per year

| Age | Urban men | Urban women | Rural men | Rural women |
| --- | --- | --- | --- | --- |
| 40-49 | 66000 (55000-76000) | 33000 (28000-38000) | 16000 (14000-19000) | 26000 (22000-30000) |
| 50-59 | 130000 (110000-150000) | 28000 (24000-33000) | 18000 (15000-20000) | 32000 (27000-37000) |
| 60-69 | 52000 (44000-61000) | 35000 (30000-41000) | 6200 (5200-7200) | 22000 (18000-26000) |

(B) Averted strokes per year

| Age | Urban men | Urban women | Rural men | Rural women |
| --- | --- | --- | --- | --- |
| 40-49 | 950 (800-1100) | 3300 (2800-3800) | 890 (740-1000) | 3700 (3100-4300) |
| 50-59 | 7000 (5900-8200) | 5600 (4700-6500) | 7800 (6500-9100) | 8500 (7100-9900) |
| 60-69 | 6000 (5000-6900) | 4100 (3400-4700) | 9000 (7500-10000) | 7000 (5900-8200) |

(C) Averted deaths per year

| Age | Urban men | Urban women | Rural men | Rural women |
| --- | --- | --- | --- | --- |
| 40-49 | 7700 (6000-9400) | 1300 (1000-1600) | 3000 (2300-3600) | 1500 (1100-1800) |
| 50-59 | 29000 (22000-36000) | 2800 (2200-3300) | 10000 (8000-13000) | 4400 (3500-5300) |
| 60-69 | 25000 (19000-30000) | 9400 (7300-11000) | 6200 (5000-7500) | 8500 (6700-10000) |
