# Supplementary material for: Dietary Salt Reduction and Cardiovascular Disease Rates in India: A Mathematical Model
Source: PLoS One. 2012 Sep 6;7(9):e44037. doi: 10.1371/journal.pone.0044037 (PMC3435319; doi:10.1371/journal.pone.0044037)
Supplement: Text S1 — Description of data sources and modeling approach. (DOC) [file pone.0044037.s017.doc]

**SI Text. Description of data sources and modeling approach**

In this Supporting Information, we provide the full input data and additional model details to permit independent replication of our results.

*Modeled population*

We modeled three age cohorts 40-49, 50-59 and 60-69 years of age, subdivided into men and women and urban versus rural location. The starting population sizes of the cohorts and the rate of entry of new people into the 40-49 year old age group were obtained from the public source file of the Indian Census Bureau (SI Figure 1) . The WHO has published cause-specific mortality trends among Indians , providing an estimate of the non-MI/non-stroke rate of death in each gender and age category. Using a standard open-source competing risks algorithm , we applied the WHO estimates to each cohort to calculate the number of non-MI and non-stroke deaths (SI Table 4). Note that in each salt reduction scenario, the application of this algorithm allows individuals “saved” by salt reduction from a MI or stroke in a given year to potentially die of other events, given the rate of non-MI/non-stroke death for their age, gender and location. Conversely, persons who do not die from, for example, tuberculosis would be susceptible to MIs and strokes when subjected to this algorithm. As shown in SI Table 5, the time-trends in these non-MI/non-stroke mortality rates were explicitly incorporated into the model, since a significant change over time is occurring among the Indian population (e.g., as infectious disease rates decline).

*MI and stroke rates*

The WHO has also calculated the rate of incident and recurrent MI and stroke per annum among Indian men and women in each age and location (rural versus urban) cohort (SI Tables 1 and 2) as well as case fatality following MI and stroke events (SI Table 3) ; case fatality is defined as the number of people who die within 30 days of an MI or stroke event.

To ensure face validity of the model, we checked that predictions from the model for each subpopulation were consistent with independent data that were not used to derive the input parameters (SI Figure 2) .

*Salt reduction scenarios*

In the salt reduction scenarios, we reduced the rate of incident MI and stroke events in the model based on a calculation of relative risk. The relative risk after salt reduction was equal to the decline in systolic blood pressure for each gram reduction in salt, multiplied by the reduction in relative risk of MI or stroke for each millimeter mercury decline in systolic blood pressure.

First, a meta-analysis of randomized trials of over four weeks duration was used to estimate the relationship between each gram reduction on salt intake and subsequent millimeters mercury of systolic blood pressure reduction . The analysis estimated a reduction of 50 mmol/day (3 g/day) in salt intake predicts a fall in systolic blood pressure of 3.6 mmHg (95% CI: 2.8 to 4.4) among hypertensives (systolic pressure >140 mmHg) and of 1.8 mmHg (95%CI: 1.0 to 2.6) among normotensives. The analysis further supported a linear dose-response relationship between salt intake reduction and systolic blood pressure reduction. The prevalence of hypertension by age cohort, gender and location (urban or rural) for the model was taken from the WHO SAGE study in India , a population-representative examination survey of 2,834 men and 3,442 women in India aged 40 to 69 (SI Table 6).

Next, the reduction in relative risk of MI and stroke from each millimeter mercury decline in systolic blood pressure was estimated by the Smith-Spangler equations , which derived the relative risk based on a meta-analysis of individual data for one million adults in 61 prospective studies . The relative risk reductions from regressions in this analysis are age-specific and are equal to 2^(decrease in systolic pressure * -0.000011009*age^2 + 0.00086305*age + 0.035176) for MI, and equal to 2^(decrease in systolic pressure * -0.000025946*age^2 + 0.0023052*age + 0.022168) for stroke, as derived elsewhere in detail . We used the Indian Census profile of the age-distribution of each cohort to produce a distribution of the relative risk in each cohort for uncertainty analysis sampling, as described below.

*Sensitivity and uncertainty analyses*

We conducted sensitivity analyses varying the impact of salt reduction on changes in cardiovascular risk. For a pessimistic bound, we reduced the benefit of lowered blood pressure such that lowering blood pressure to a given level conferred only two-thirds of the benefit received by a person with native blood pressure at that lower level . For an optimistic bound, we simulated the greater blood pressure reduction achieved per gram salt reduction among older adults according to some clinical trials (in which the change in systolic pressure = -0.0598 * (mmol salt reduction) - 0.0431 * (age-48)) , as opposed to the more conservative meta-analysis relationship used in the baseline simulation .

Additional multivariate uncertainty analyses were also performed. Specifically, Monte Carlo simulations were used to estimate the uncertainty around outcomes. Normal distributions were constructed around the means and variances of each of the model parameters as displayed in the SI Tables. For each simulated salt reduction scenario, we sampled from the distributions 10,000 and report the mean and 95% CI around the model output. Samples were drawn independently from all distributions. All model calculations were performed in MATLAB version R2011b (MathWorks, Cambridge).

In the manuscript, we report these results for the 3 g/day reduction in dietary salt achieved over 30 years from 2011 to 2041. Here, we additionally display a series of figures showing the impact of varying this target to 1 g/day and to 4 g/day, achieved over the same 30-year time period (SI Figures 5 and 6).

**References**

1. Registrar-General of India. Census Data. Delhi: Office of the Registrar-General, 2011.

2. Global Mortality Database [database on the Internet]. WHO. 2010.

3. The R Foundation for Statistical Computing. Mstate Package. CRAN; 2011.

4. Shah B, Kumar N, Menon G, Khurana S, Kumar H. Assessment of burden of non-communicable diseases. Delhi: WHO India, 2010.

5. He FJ, MacGregor GA. Effect of longer-term modest salt reduction on blood pressure. Cochrane Database Syst Rev. 2004(3):CD004937. Epub 2004/07/22.

6. World Health Organization. Study on global AGEing and Adult Health (SAGE). Geneva: WHO, 2011.

7. Smith-Spangler CM, Juusola JL, Enns EA, Owens DK, Garber AM. Population strategies to decrease sodium intake and the burden of cardiovascular disease: a cost-effectiveness analysis. Annals of internal medicine. 2010;152(8):481-7, W170-3. Epub 2010/03/03.

8. Lewington S, Clarke R, Qizilbash N, Peto R, Collins R. Age-specific relevance of usual blood pressure to vascular mortality: a meta-analysis of individual data for one million adults in 61 prospective studies. Lancet. 2002;360(9349):1903-13. Epub 2002/12/21.

9. Law MR, Frost CD, Wald NJ. By how much does dietary salt reduction lower blood pressure? III--Analysis of data from trials of salt reduction. BMJ. 1991;302(6780):819-24. Epub 1991/04/06.

10. Sacks FM, Svetkey LP, Vollmer WM, Appel LJ, Bray GA, Harsha D, et al. Effects on blood pressure of reduced dietary sodium and the Dietary Approaches to Stop Hypertension (DASH) diet. DASH-Sodium Collaborative Research Group. The New England journal of medicine. 2001;344(1):3-10. Epub 2001/01/04.

11. MacGregor GA, Markandu ND, Sagnella GA, Singer DR, Cappuccio FP. Double-blind study of three sodium intakes and long-term effects of sodium restriction in essential hypertension. Lancet. 1989;2(8674):1244-7. Epub 1989/11/25.

12. International Institute for Population Sciences. National Family Health Survey, India. Mumbai: IIPS, 2000.
